# Supplementary material for: The histone chaperones Vps75 and Nap1 form ring-like, tetrameric structures in solution
Source: Nucleic Acids Res. 2014 Mar 31;42(9):6038–51. doi: 10.1093/nar/gku232 (PMC4027167; doi:10.1093/nar/gku232)
Supplement: SUPPLEMENTARY DATA [file supp_42_9_6038__index.html]

The histone chaperones Vps75 and Nap1 form ring-like, tetrameric structures in solution — The histone chaperones Vps75 and Nap1 form ring-like, tetrameric structures in solution — The histone chaperones Vps75 and Nap1 form ring-like, tetrameric structures in solution — SUPPLEMENTARY DATA 

# The histone chaperones Vps75 and Nap1 form ring-like, tetrameric structures in solution

## SUPPLEMENTARY DATA

**Files in this Data Supplement:**

- SUPPLEMENTARY DATA
