# Supplementary material for: The histone chaperones Vps75 and Nap1 form ring-like, tetrameric structures in solution
Source: Nucleic Acids Res. 2014 Mar 31;42(9):6038–51. doi: 10.1093/nar/gku232 (PMC4027167; doi:10.1093/nar/gku232)

Figure S1. De novo generated bead models from small angle X-ray scattering of the Vps75 tetramer. The program DAMMIN was used to generate bead models from X-ray scattering data collected from Vps75 at 150mM. Many of the models generated show either a hole or a deep depression within the centre of the structure. For comparison, the Vps75 tetramer model generated from PELDOR measurements is shown in the bottom right corner.

Figure S2. The triple mutant of Vps75 R164D K169E and K170D prevents Vps75 tetramerisation. SEC-MALS elution profiles of wild type Vps75 (blue) and Vps75 R164D K169E K170E (red) at 150 mM sodium chloride.

Figure S3. The cross-linking efficiency of Vps75 K78C is diminished under non-tetramerising conditions. (A) SDS-PAGE analysis of Vps75 K78C (1.25 µM, 25 pmoles) disulphide cross-linked across the tetramerisation interface with a 2-fold increasing titration of Copper (II) Phenanthroline (CuP) from 2.5 to 40 pmoles for 2 minutes at room temperature under conditions which favour a Vps75 tetramer (150 mM NaCl) or a dimer (500 mM NaCl). (B) SDS-PAGE analysis of Vps75 K78C (1.25 µM, 25 pmoles) titrated with increasing concentrations of $(H3H4)_2$ (5, 10, 15, 20, 25, 50 pmoles) and disulphide cross-linked with CuP (20 pmoles) at 150 mM NaCl for 2 minutes at room temperature.

Table S1. Summary of Vps75 PELDOR data compared to the distances extracted from the XPLOR-NIH generated model.

Bowman and Hammond et al., Table S1

Labelling site	Distance	EPR distance / Å	Used for modelling	Model distance / Å	Deviation / Å
Y35Rx2	AB	78.00	Y	79.70	1.70
E56R1	AB$^{¥}$	71.00	N	69.10	-1.90
E56R1	AC	33.00	Y	34.14	1.14
E56R1	AD	74.00	N	67.20	-6.80
K117R1	AB$^{¥}$	85.00	N	90.20	5.20 *
K117R1	AC	ND	N	37.03	-
K117R1	AD	91.00	N	94.41	3.41 *

¥ AB distances are quoted from the high salt PELDOR data
* Intra-dimer AB distance deviates from crystal structure at high salt which has a knock on effect on the AC and AD distances
ND – Not determined

Table S2

Mutant	Retention Time (min)
wt Vps75 Tetramer	14.69
wt Vps75 Dimer	16.75
D162K	14.57
K161D	14.77
K173E	14.85
K171E	14.85
E218R	14.96
E224R	15.07
K163E	15.39
D225R	15.42
K177E	15.46
R73E	15.53
K170E	15.67
K169E	15.72
R164D	15.85

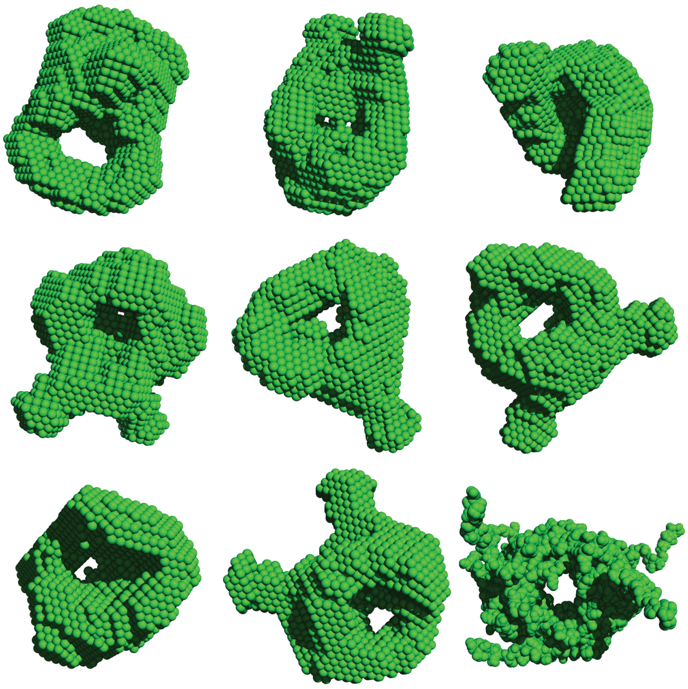

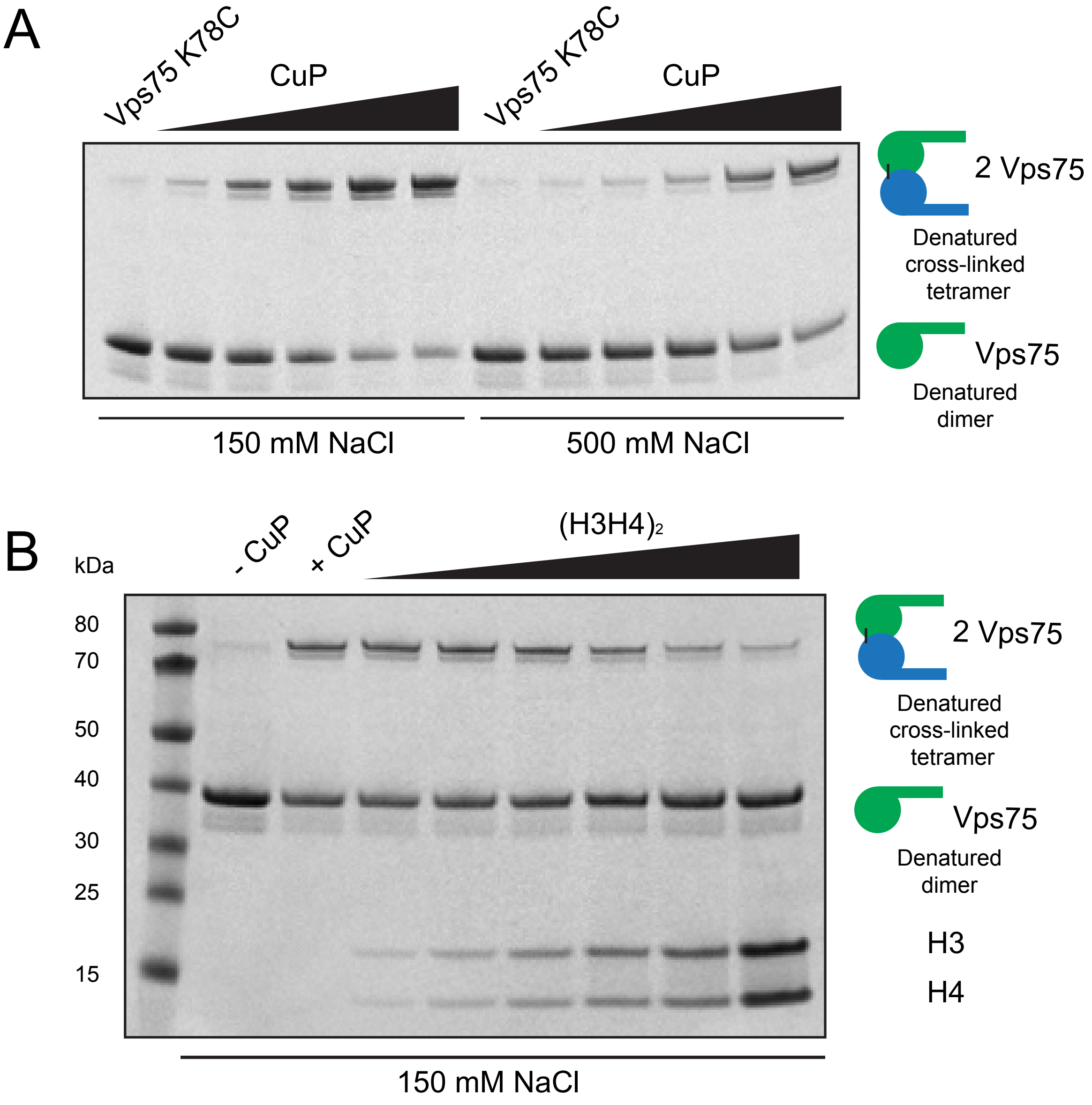
A
Vps75 K78C
CuP
Vps75 K78C
CuP
2 Vps75
Denatured cross-linked tetramer
Vps75
Denatured dimer
150 mM NaCl
500 mM NaCl
B
kDa
- CuP
+ CuP
$(H3H4)_2$
80
70
50
40
30
25
15
2 Vps75
Denatured cross-linked tetramer
Vps75
Denatured dimer
H3
H4
150 mM NaCl

Bowman and Hammond et al., Figure S3

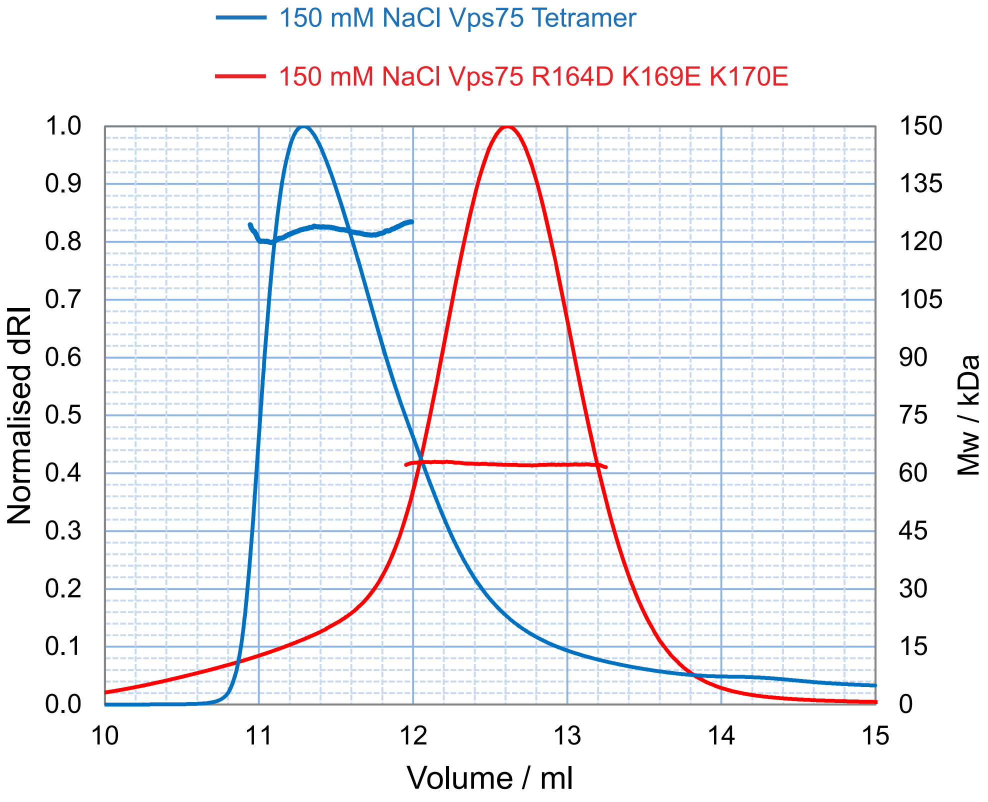

Supplement: SUPPLEMENTARY DATA [file supp_gku232_nar-02436-m-2013.zip › nar-02436-m-2013-File010.pdf]
