# Supplementary material for: The histone chaperones Vps75 and Nap1 form ring-like, tetrameric structures in solution
Source: Nucleic Acids Res. 2014 Mar 31;42(9):6038–51. doi: 10.1093/nar/gku232 (PMC4027167; doi:10.1093/nar/gku232)
Supplement: SUPPLEMENTARY DATA [file supp_gku232_nar-02436-m-2013.zip › nar-02436-m-2013-File001.doc]

**The histone chaperones Vps75 and Nap1 form ring-like, tetrameric structures in solution**

Andrew Bowman^1,^, Colin M. Hammond^1^, Andrew Stirling^1^, Richard Ward^2^, Weifeng Shang^3^, Hassane El-Mkami^4^, David A.Robinson^5^, Dmitri I. Svergun^3^, David George Norman^2^ and Tom Owen-Hughes^1^*.

^1^ Centre for Gene Regulation and Expression, University of Dundee, Dundee, DD1 5EH, UK.

^2^ Nucleic Acids Structure Research Group, University of Dundee, Dundee, DD1 5EH, UK.

^3^ European Molecular Biology Laboratory, Hamburg Outstation, c/o DESY, Notkestrasse 85, D-22603 Hamburg,Germany.

^4^ School of Physics and Astronomy, University of St Andrews, St Andrews FE2 4KM, UK.

^5^ Division of Biological Chemistry and Drug Discovery, University of Dundee, Dundee, DD1 5EH, UK.

* To whom correspondence should be addressed. Tel: 44 1382 385796; Fax: 44 1382 388702; Email: t.a.owenhughes@dundee.ac.uk

"The authors wish it to be known that, in their opinion, the first 2 authors should be regarded as joint First Authors".

Present Address: Andrew Bowman, Department of Physiological Chemistry, Adolf-Butenandt-Institute, Butenandtstrasse 5, 81377 Munich, Germany. Weifeng Shang, Centre for Radiation Research and Instrumentation, Department of Biological, Chemical and Physical Sciences, Illinois Institute of Technology, 3101 S.Dearborn, Chicago IL. 60616, USA.

**ABSTRACT**

NAP-1 fold histone chaperones play an important role in escorting histones to and from sites of nucleosome assembly and disassembly. The two NAP-1 fold histone chaperones in budding yeast, Vps75 and Nap1, have previously been crystalized in a characteristic homodimeric conformation. In this study a combination of Small Angle X-ray Scattering, Multi Angle Light Scattering and Pulsed Electron-electron Double Resonance approaches were used to show that both Vps75 and Nap1 adopt ring shaped tetrameric conformations in solution. This suggests that the formation of homotetramers is a common feature of NAP-1 fold histone chaperones. The tetramerisation of NAP-1 fold histone chaperones may act to shield acidic surfaces in the absence of histone cargo thus providing a ‘self-chaperoning’ type mechanism.

.

**Introduction**

Histone chaperones were first characterised as factors required for correct assembly and disassembly of nucleosomes during DNA replication . More recently, histone chaperones have been shown to be involved in many other aspects of chromatin dynamics, functioning both with ATP driven molecular motors and post-translational modifiers in diverse processes such as transcriptional regulation, DNA repair, replication, homologous recombination, and mRNA maturation (reviewed in ).

Vps75 was originally identified in a genetic screen to isolate factors affecting vacuolar protein sorting in budding yeast , but was re-classified as a NAP-1 family histone chaperone based on sequence homology . High affinity for both H3-H4 and H2A-H2B confirmed the histone chaperone function of Vps75 . Vps75 co-purifies with the histone H3 acetyl-transferase Rtt109 , and has been reported to increase efficiency of its histone acetylation activity by over 250 fold . The catalysis of histone acetylation by chaperone dependent histone acetyl transferase complexes reveals interconnectivity between the regulation of chromatin organisation and nucleosome assembly/disassembly pathways.

The presence of near stoichiometric amounts of Vps75 and Rtt109 after stringent purification of the tagged complex suggests that the interaction between the two proteins is stable . However, genome-wide genetic interaction analysis has revealed distinct cellular roles for Vps75 and Rtt109: Rtt109 functioning in DNA replication and repair pathways, and Vps75 having an additional function in transcriptional regulation . These distinct roles may explain the alternate expression patterns of Rtt109 and Vps75. Whilst Rtt109 transcripts fluctuate during the cell cycle, peaking during S-phase, Vps75 transcripts protein levels remain relatively stable . Thus, it has been suggested that a Rtt109 free pool of Vps75 exists outside of S-phase .

Structural analyses of Vps75 reveals the protein to be a homodimer , adopting the ‘headphone’ fold common to all Nap1-like proteins . Subsequent analyses of Vps75 bound to Rtt109, led to the discovery of two distinct structures involving the binding of either one or two Rtt109 molecules per Vps75 dimer . Although the stoichiometries of the two characterised constructs alter, the interaction surfaces between Rtt109 and Vps75 in both structures are similar. The physiological relevance of the two alternate structures has yet to be established (reviewed by ).

The structurally related yeast protein Nucleosome Assembly Protein 1 (Nap1) shares many characteristics of Vps75 in that it interacts with all four core histones and has also been implicated in transcriptional regulation . Unlike Vps75, Nap1 has been shown to form higher order assemblies dependent both on the ionic environment and its concentration. Whereas Nap1 is predominantly dimeric at high salt concentrations, independent of its concentration, at lower salt concentrations the protein self associates to form soluble aggregates in a concentration dependent manner . However, the exact nature of these self associated states of Nap1 is still unclear, although it has been suggested to involve a beta-hairpin loop that protrudes from the core of the protein, and is capable of making beta sheet structures with neighboring Nap1 molecules .

In this study, we report that Vps75 adopts a homo-tetrameric conformation in solution at low salt concentrations. Furthermore, we derive a molecular model for the solution structure of Vps75 in its tetrameric conformation through a combination of pulsed electron double resonance (PELDOR), small angle X-ray scattering (SAXS) and structure guided mutagenesis. In addition, our findings using multi angle light scattering (MALS), and PELDOR indicate that the structurally related protein Nap1 also adopts a discrete tetrameric conformation under physiological ionic conditions.

**MATERIAL AND METHODS**

**Recombinant protein expression and purification**

The open reading frame of Vps75 was cloned into a modified pET30a vector carrying a six histidine N-terminal tag followed by a tobacco etch virus (TEV) protease cleavage site. The two native cysteine residues at positions 21 and 212 were mutated to alanine by PCR based mutagenesis. For site directed crosslinking spin labelling a unique cysteine was engineered at position Tyr35. For site directed spin labeling, unique cysteine residues were engineered at position Lys117 and Glu56. Rtt109 was expressed from a pET28a derived vector, a kind gift from Paul Kaufman. Protein was expressed in *E. coli* strain BL21 DE3 pLysS (Stratagene) and purified by a combination of cobalt affinity, ion exchange and gel filtration chromatography . For mutagenic analysis of Vps75 tetramerisation, point mutants were created in the C21A/C212A construct and isolated using a single step cobalt affinity purification. The open reading frame of Nap1 was cloned into a pET15b vector (Novagen) and the cysteine residues 200, 249, 272 and 414 were mutated to alanine. For site directed spin labelling experiments, unique cysteine residues were engineered at E209, T251 and T307.

**Deuterated protein expression and purification**

*E. coli* (strain BL21 DE3 pLysS (Stratagene)) carrying Vps75 Y35C, K117C or E56C in a modified pET30a expression vector or Nap1 E209C, T251C or T307C in a pET15b expression vector were grown in 20 mL of LB until saturation. Cells were collected by centrifugation and washed twice in 1 mL of Spectra9 fully deuterated media (CIL) and resuspended in a final volume of 200 mL of the same media. Cells were grown to an OD_600nm_ of 0.7 at 37 ⁰C (roughly 8 hours of growth), induced with 0.5 mM IPTG at 30 ⁰C for 18 hours. Cells were collected and lysed by freeze-thaw followed by sonication in 20 mM Tris-HCL pH8.5, 150 mM NaCl supplemented with protease inhibitors. Clarified lysate was bound to 0.5 mL of HisPur cobalt affinity resin (Thermo Scientific), washed extensively and eluted in the same buffer with 200 mM imidazole added. Eluted protein was incubated overnight with TEV protease and 5 mM dithiothreitol, to remove the N-terminal six histidine tag, and further purified by gel filtration using a Superdex S200 GL 10/300 column (GE Healthcare) equilibrated with 20 mM HEPES-KOH and 0.5 M sodium chloride. Peak fractions were pooled, concentrated using centrifugal concentrators (Amicon Ultra 4 MWCO 10000, Millipore) and stored at -80 ⁰C. The extent of deuteration was assessed by MALDI-TOF mass spectrometry and found to be virtually 100% deuterated in all cases (data not shown).

**Size exclusion chromatography and multi angle light scattering (SEC-MALS)**

SEC-MALS experiments were performed on a Dionex Ultimate 3000 HPLC system with an inline Wyatt miniDAWN TREOS multi angle light scattering detector and Optilab T-rEX refractive index detector. Initially SEC-MALS experiments were performed on a MAbPac SEC– 1 (Dionex) column (Figure 1) and due to column degradation subsequent experiments were performed on a Superdex S200 CL 10/300 (GE Healthcare) column (Figure 5 and 7). Buffer conditions were 20 mM HEPES-KOH pH 7.5 with sodium chloride concentrations as stated in the text. Molar masses spanning elution peaks were calculated ASTRA v6.0.0.108 (Wyatt).

**Bio-layer Interferometry**

Bio-layer interferometry experiments were performed using dip and read technology on an Octet® RED384 System (ForteBio) using Streptavidin (SA) biosenors. Following purification, Nap1 fold chaperones were biotinylated in a site specific manner (Vps75 Y35C and Nap1 L120C) with a 5 fold excess of Maleimide-PEG2-Biotin (Thermo Scientific) overnight on ice. Unreacted Maleimide-PEG2-Biotin was removed via gel filtration on a Superdex S200 10/300 column (GE Healthcare). Biosensors were equilibrated in buffer (1 M NaCl, 20 mM HEPES.KOH pH 7.5) for 60 seconds and subsequently loaded with either biotinylated Nap1 or Vps75 (60 µg/ml) in the same buffer for 600 seconds. Free streptavidin binding sites were blocked with biocytin (10 µg/ml) for 60 seconds and biosensors were washed in fresh equilibration buffer for 120 seconds. Subsequently the biosensor was equilibrated in the binding buffer (150 mM NaCl, 20 mM HEPES.KOH pH 7.5 with 0.1% BSA) for 120 seconds prior to starting the binding assay. All subsequent measurements were performed in the aforementioned binding buffer. During the assay the association of unlabeled wild type chaperone with its counterpart immobilised on the biosensor was measured for 120 seconds and the dissociation measured by dipping into fresh binding buffer for 120 seconds. Experiments were performed in triplicate across a 3 fold dilution concentration range of unlabeled chaperone as specified in Figures 1C and 7C. Data output by the ForteBio data analysis software v. 7.0 was plotted in SigmaPlot 12.0. Steady state analysis of the concentration vs response data was performed in SigmaPlot 12.0 by fitting to a one site saturation ligand binding curve as per inset formula in Figures 1D and 7D.

**SDSL snd SDXSL**

Purified Vps75 and Nap1 with unique cysteine residues, as stated in the text, were reduced with 20 mM dithiolthrietol for 30 minutes at room temperature. The dithiothrietol was removed by gel filtration on a Superdex S200 GL 10/300 column (GE Healthcare) run in 20 mM HEPES-KOH pH 7.5, 0.5 M sodium chloride. Protein containing fractions were combined and concentrated using an Amicon Ultra centrifugal concentrator MWCO 10,000 (Millipore) to 50 μM. For SDXSL of Vps75 Y35C, the bi-functional crosslinker spin label 3,4-Bis-MTSL (Toronto Research Chemicals Inc.) was dissolved in dimethyl sulfoxide and titrated against the reduced cysteine pair in 0.2 molar equivalents, up to a final ratio of 1 molar Vps75 to 1.2 molar 3,4-bisMTSL, creating the crosslinked spin labeled sample Vps75 Y35RX2. After each addition the reactions were vigorously mixed and allowed to proceed at 25 ⁰C for three minutes before continuing with the next addition. The efficiency of labeling was assessed by separation of crosslinked from non-crosslinked species by SDS-PAGE and coomassie staining. A labeling efficiency of 90% was typically achieved. For SDSL of Vps75 K117C and E56C and Nap1 E209C, T251C and T307C, a ten-fold excess of MTSL was added to the pooled peak fractions and labelling left to proceed for 20 minutes at 25 ⁰C, before dialysis overnight at 4 ⁰C against the same buffer to remove all traces of unreacted MTSL. Labeled protein was then exchanged to buffer containing deuterium oxide (Sigma-Aldrich) in place of water, double the final sodium chloride concentration (as mention in the text) and 20 mM HEPES-KOH pH 7.5, using centrifugal concentrators (Millipore). After four rounds of exchange, protein was concentrated to 200-300 μM dimer, mixed 1:1 with D8-glycerol (CIL), giving a final concentration of 100-150 μM dimer, in a final volume of 100 μL and stored at -80 ⁰C until PELDOR measurements were made.

**Pulsed Electron Double Resonance**

PELDOR experiments were conducted as described previously using a Bruker ELEXSYS E580 spectrometer operating at X band with a dielectric ring resonator and a Bruker 400U second microwave source unit. Data analysis was carried out using the DeerAnalysis 2013 package . The experimentally obtained time domain traces were first adjusted to remove background decay. Tikhonov regularisation was then used to obtain the most appropriate distance distributions from each dataset.

**Modelling the structure of the Vps75 tetramer.**

Refinement of the Vps75 tetramer structure was carried out using XPLOR-NIH . Spin labels were added either as random coordinates and then regularised using molecular dynamics with the rest of the protein held fixed, or alternatively MTSL was added using the Pymol plugin MTSSLWizard . From both of these approaches ensembles of spin-label conformations were obtained that sampled the available space. The nitrogen atoms, of each spin label in each ensemble of conformations were incorporated into the coordinate file as single residues. Distance restraints determined from the distance distributions measured by PELDOR were incorporated as Average=R-3 restraints between label ensembles and rigid body dynamics was used to minimise the distance restraint violations. Symmetry constraints were used to ensure that the overall symmetry of the Vps75 tetramer contacts were preserved. Multiple refinement runs were carried out starting from separated Vps75 dimer arrangements. Final structures with high distance restrain violations were discarded and low violation structures retained. For information on the distances restraints used to model the Vps75 and deviations observed in the final models see Table S1.

**Small Angle X-ray Scattering (SAXS)**

The SAXS measurements for the C-terminal truncated Vps75 and the full length Vps75 under low (150 mM NaCl) and higher salt concentrations (500 mM NaCl) were made at the EMBL BioSAXS beamline X33 at the DORIS storage ring, DESY (Hamburg, Germany). A photon counting PILATUS 1M detector was used to record the scattered X-ray at a wavelength of 1.54 Å. The sample-to-detector distance was 2.7 m, yielding a maximum recordable momentum transfer (s = 4π sinθ/λ) of 0.5 Å^-1^. A bovine serum albumin solution at 4.5 mg/ml in 50 mM Hepes pH 7.5 was used for calibrating the molecular mass. A series of concentrations bewteen 0.5 and 10 mg/ml was measured for each sample at 10 °C and no concentration dependence of the scattering profiles was observed for all samples. The standard SAXS data reduction and analysis were done using PRIMUS . The pair distance distribution function *p(r)* and maximum size of the protein molecules *D_max_* was calculated with the program GNOM . The program CRYSOL was used to calculate the theoretical X-ray scattering profile with the high-resolution model from crystal structures. Missing termini were represented by a chain of dummy residues and added to the crystal structures using the program BUNCH to best fit the experimental data from full length Vps75.

**RESULTS**

**Two dimers of Vps75 self associate to form a homo-tetramer.**

Like all Nap1 related proteins characterised to date, Vps75 dimerises, however, higher order associations of this chaperone have yet to be reported. During biochemical analysis of Vps75 by gel filtration chromatography we discovered that its elution profile changes depending on the ionic conditions employed. At 150 mM sodium chloride, Vps75 elutes significantly earlier during size exclusion chromatography than at high salt concentrations indicative of Vps75 oligomerisation beyond the dimeric form (Figure 1A). To identify the oligomeric form of Vps75 present under these two conditions Size Exclusion Chromatography coupled to Multi Angle Light Scattering (SEC-MALS) was employed for molecular weight determination. Under the stringent conditions of 500 mM sodium chloride, Vps75 eluted with an average molecular weight of 60.1 kDa (Figure 1A), close to the theoretical mass of 61.2 kDa calculated for a dimer. At 150 mM sodium chloride, Vps75 eluted with an average molecular weight of 117.9 kDa, close to the theoretical molecular weight of two dimers, 122.4 kDa. Thus, it appears that two dimers of Vps75 can associate to form a tetramer in a manner dependent on electrostatic interactions.

Vps75 contains three major domains: the structurally defined dimerisation and earmuff domains and an acidic C-terminal domain thought to be devoid of stable secondary and tertiary structure . We were interested to know whether the interaction between two Vps75 dimers was mediated by the folded, globular region or the disordered C-terminal region of Vps75. To address this we created a truncated version of Vps75 lacking the C-terminal acidic region. This construct was analysed by gel filtration coupled to MALS and found to display the same characteristics of the full length protein (Figure 1B), eluting with an average molecular weight of 56.2 kDa (theoretical dimer – 54 kDa) and 110.9 kDa (theoretical tetramer – 108 kDa), in stringent and physiological conditions respectively. We therefore concluded that interactions mediating tetramerisation must involve the structurally defined core of the protein.

In order to assess the concentration dependence of Vps75 self association, Vps75 Y35C dimers were specifically labelled with Maleimide-PEG2-Biotin which provided a means of attachment to Streptavidin biosensors. After attachment and washing, association of unlabelled Vps75 was measured by bio-layer interferometry, and used to calculate a *K_d_* for tetramerisation of 0.6 uM (Figure 1CD).

**Analysis of the tetrameric structure of Vps75 in solution by SDXSL and PELDOR**

Vps75 has previously been crystallised as a homodimer . In order to characterise the tetrameric conformation we detected in solution, we set out to obtain distance measurements within the complex using PELDOR in conjunction with Site-Directed Spin Labelling (SDSL). The traditional approach for SDSL utilises a thiol reactive nitroxide compound attached to a unique cysteine residue that is engineered at a location of choice through genetic manipulation of the coding sequence. When analysing a homodimeric complex a single cysteine residue per polypeptide chain results in a doubly labelled system. With regards to homotetrameric structures, a one-chain-one-label approach results in four spins per protein complex. Such label dense systems are often difficult to deconvolute, as multiple distances are encoded within a single spectrum. To avoid this and maintain a two spin system, we investigated the possibility of singly labelling a dimer through crosslinking of two cysteine residues using the bi-functional crosslinking spin label 3,4-Bis-(methanethiosulfonylmethyl)-2,2,5,5-tetramethyl-2,5-dihydro-1H-pyrrol-1-yloxy radical, or 3,4-Bis-MTSL (Figure 2A). We scanned Vps75 for a residue that was both solvent accessible and came into close proximity with its partner residue in the opposing dimer. The tyrosine at position 35 fulfilled these criteria and reacted well with 3,4-Bis-MTSL, crosslinking to a high efficiency (Figure 2AB). Specificity of crosslinking across the dimerisation interface was assessed by gel filtration chromatography (data not shown). Whilst this research was being carried out, a similar approach was reported that used the same compound to label two adjacent cysteine residues, but on the same polypeptide chain . To highlight the difference in crosslinking labelling across a dimeric interface as a means to singly label a protein dimer, we named this spin labelled side chain 'RX2', and this alternate form of spin labelling Site-Directed crosslink (X) Spin Labelling (SDXSL) (Figure 2A).

We considered three modes via which Vps75 dimers could interact to form tetramers (Figure 2C). Firstly through interactions solely involving the earmuff domains in a side-by-side configuration termed ‘M’. This model seems unlikely as it does not suggest a mechanism whereby oligomerisation of Vps75 is curtailed at the tetrameric level. In a second model, tetramerisation could form a ring structure through contacts between the earmuff domains termed ‘O’. In a third model, the antiparallel helices of the dimerization domain could form a 4 helix bundle or ‘X’ structure. Initial EPR measurements with Vps75 Y35RX2 under tetramerising conditions revealed the presence of a long distance in excess of 7 nm (data not shown). Such large distances are difficult to define due to the traverse relaxation of the excited state of the spin label radical electrons by the nuclear spins of surrounding protons. This detrimentally affects the timing window over which the raw dipolar evolution function can be measured. Recently, we have shown that complete deuteration of proteins can significantly extend the relaxation time T_m_ allowing dipolar interactions between the spin labels to be measured over a longer time period, thereby allowing measurement of much larger distances . Indeed, deuteration of Vps75 Y35RX2 extended the timing window over which the dipolar evolution function could be measured to 15μs which was sufficient to observe the full spin echo oscillation (Figure 2D). Upon Tikhonov regularisation of the background corrected dipolar evolution function to a distance distribution (Figure 2E), a modal distance of 7.8 nm between the two cross-linked Y35RX2 residue pairs in the Vps75 tetramer was extracted (Figure 2F).

Although long enough to rule out the ‘X’ model, 7.8 nm between RX2 labels could still be satisfied by both the ‘M’ models and ‘O’ models. The ‘M’ model was rejected in favour of the ‘O’ model which provides a more obvious mechanism of termination of Vps75 oligomerisation at the tetrameric level as observed by SEC-MALS (see below). Initial attempts to define a structural model of the Vps75 tetramer using the crystal structure of the Vps75 dimer in a ring-like orientation revealed the extracted distance between Y35RX2 residues could be met by a limited number of sterically allowed dimer-dimer orientations rotated around an axis drawn between the two Y35RX2 labels. Thus further constraints were sought to confirm and refine the rotational position of the two Vps75 dimers relative to each other.

**Refinement of the Vps75 tetramer structure using a four-spin system**

In order to define the rotational relationship between the Vps75 dimers, labelling sites on the earmuff domains were chosen with the aim of defining the short distance between earmuffs across the tetramerisation interface. Such a labelling strategy results in a four-spin system from which it is often difficult to precisely interpret PELDOR data. A four spin system consisting of spins a, b, c and d, with two perpendicular planes each possessing two-fold rotational symmetry (that of the dimer and that of the tetramer), will result in distances AB, CD and AD (Figure 3A). The distance across the dimer interface was denoted AB (which is equivalent to the distance CD). The distances across the tetramer interface were denoted AC and AD (which are equivalent to BD and BC, respectively). From initial modelling attempts a strategy was devised to probe labelling sites on the earmuff domains of Vps75 that would most likely produce short AC distances that were resolvable from longer AB and AD distances (Figure 3A). Thus, whilst absent in the dimer, upon tetramerisation these shorter distances should be easily identifiable. Labelling sites at positions K117 and E56 were chosen for this purpose.

The intradimer distance, AB, for both K117R1 and E56R1, was assigned by carrying out PELDOR under high salt conditions where Vps75 is in its dimeric form (Figure 3B and C). Protein deuteration allowed the measurement of distances close to 8.5 nm for K117R1 and 7.3 nm for E56R1, to be extracted, as was expected from current crystal structures of the Vps75 dimer. Carrying out the same experiment under conditions that favour tetramer formation revealed distinct changes in the dipolar evolution for both sites (Figure 3B and C). Changes in the modulation depth indicated a four spin system , as expected, for both labelling sites, with the oscillation becoming more convoluted compared to that of the dimer. Tikhonov regularisation revealed additional unique distances (Figure 3C). In both cases a well resolved shorter distance distribution was present, which was assigned to the theoretical distance AC. The K117R1 labelling site produced an additional long distance of 9.1 nm, which was assigned the theoretical distance AD (Figure 3C). The distance AD was not as well resolved for E56R1, and most likely overlaps quite closely with the distance AB (Figure 3C).

Using the additional distances gained from the four spin system, a molecular model of the Vps75 tetramer was produced using the crystal structure of the dimer (PDB code: 2ZD7) as a building block. Spin labels were built onto the atomic structure of the Vps75 and molecular dynamic simulations were carried out to identify likely conformational ensembles for each nitroxide labelling site. Rigid body minimisation, using PELDOR distance measurements (Table S1) and non-crystallographic symmetry restraints, was then used to refine the structure of the tetramer. The resulting model indicates that interactions between the earmuff domains contribute to the tetramerisation interface with a two-fold plane of symmetry running through the earmuff-earmuff interaction interface of alternate dimers, resulting in rotation of the dimerisation domains by ~60 degrees with respect to each other (Figure 3D).

**SAXS analysis of the Vps75 tetramer**

To further validate the model of the Vps75 tetramer observed in SEC-MALS and EPR experiments, Small Angle X-ray Scattering (SAXS) experiments were carried out. Guinier analysis of the SAXS data for C-terminal truncated Vps75 under high salt concentrations (500 mM NaCl) yielded a radius of gyration (R_g_) of 32.5±0.5 Å and a molecular weight (MW) of 60±5 kDa, which is approximately twice the MW estimated from the protein sequence of a Vps75 monomer, 26.5 kDa. In comparison the CRYSOL calculation of the corresponding atomic model of the Vps75 dimer (PDB accession code: 2ZD7) yields a consistent value of R_g_ = 31.8 Å. A direct comparison of the experimental scattering profile with that calculated from the crystal structure gives a good fit with discrepancy χ=1.4 (data not shown). These results confirm that the C-terminal truncated Vps75 molecule forms a stable dimer in solutions containing high salt concentrations and the conformation observed in the crystal structure (PDB code: 2ZD7) is preserved.

The M_W_ of the full length Vps75 assemblies estimated from SAXS analysis is 65±5 and 120±10 kDa for high and low salt concentrations respectively. This is in close agreement with the theoretical molecular weights of a dimer, 61.2 kDa, and tetramer, 122.4 kDa and consistent with the SEC-MALS analysis. As expected from the M_W_ values, the change of ionic conditions leads to a dramatic change in structure of the Vps75 solute, evidenced by the markedly different scattering curves and distance distribution functions p(r) (Figure 4). The most probable distance (maximum of the p(r) function) of Vps75 increases from 41 Å to 52 Å (Figure 4A) upon tetramerisation and at smaller distances a close to linear increase in p(r) is observed which is typical of hollow particles. Moreover, the maximum size of the tetramer (*D_max_*=12 nm) increases only marginally compared to the dimer (*D_max_*=11 nm), and this clearly favours ‘O’ model. Furthermore, bead models that fit the observed scattering were frequently observed to have a ring-like organisation (Supplementary Figure S1). Indeed the increase of the maximum size would be larger if Vps75 were to adopt the ‘M’-like structure.

In order to account for the unstructured C-terminal tails in full-length Vps75 assemblies the C-termini were modeled as chains of dummy residues added to the crystal structure of Vps75 using the program BUNCH . The excellent agreement between the calculated scattering curve of the resultant model of the full-length Vps75 dimer and the experimental curve is illustrated in Figure 4b (χ=1.41). The calculated scattering curve of the Vps75 tetramer with the added C-terminal residues is also in close agreement with the experimental scattering curve (χ=2.53, Figure 4c) and as such provides independent verification that the structure derived from the PELDOR distance restraints serves as a good representation of the Vps75 tetramer. The small but significant increase in the R_g_ value from 34.5±0.3 Å in high salt to 38.2±0.1 Å in low salt conditions is in line with CRYSOL predicted values of 34.6 Å and 40.4 Å calculated for the model of Vps75 with tails in a dimeric and tetrameric assembly respectively.

**Structure guided mutagenesis identifies residues at the tetramerisation interface.**

Close inspection of the tetramer model revealed extensive charge complementarity between interacting earmuff domains. An acidic patch on one earmuff domain appeared to interact with a basic patch of another in an orthogonal fashion, providing a basis for the sensitivity of Vps75 to high ionic strengths. To further test the model we generated 13 charge reversal point mutations within this region and tested their ability to tetramerise through gel filtration chromatography (Table S2). A number of these mutants significantly affected the elution profile of Vps75 and were found to cluster in the 8^th^ alpha helix of Vps75 and the preceding loop (R164D, K169E and K170E). The 3 mutations that most strongly affected elution were further characterised by SEC-MALS analysis under the tetramerising conditions of 150 mM sodium chloride (Figure 5A). The molecular weight of the mutants, Vps75 R164D (73.2 kDa), K169E (67.8 kDa) and K170E (71.9 kDa), suggests that the equilibrium for tetramerisation is significantly shifted towards the dimeric form. As such these mutants were termed non-tetramerising mutants. A triple mutant protein (R164D, K169E, K170E) also elutes with the mass of a dimer at under low salt conditions (Figure S2).

Upon further inspection of the tetramer model derived from EPR measurements, the residue K78 appeared to come into close proximity with itself on the opposite dimer Vps75 within the ring-like tetramer. By mutating K78 to cysteine and using redox potential of Cu (II) Phenanthroline as a catalyst to oxidise the cysteine residues, a disulphide bond was formed at 150 mM sodium chloride across the tetramerisation interface (not shown) demonstrating that indeed this residue does come into contact with itself in the opposing dimer. Furthermore, the presence of the induced disulphide effectively stabilised Vps75 tetramers which elute from a size exclusion column with the mass anticipated for a tetramer even under the high salt conditions that dissociate uncross-linked tetramers into dimers (Figure 5B). In addition the efficiency of cross-linking at this site was reduced at high salt concentrations that favour the presence of dimers (Figure S2A). This provides further validation that the tetramerisation interface has been defined correctly.

We also analysed the effect of adding histones to Vps75 on its ability to form cross-linked tetramers. The efficiency of Cu (II) phenanthroline cross-linking of Vps75 K78C was reduced upon titration with histones H3-H4 (Figure S2B). This suggests that Vps75, in the tetrameric conformation described here, does not bind histones H3-H4. However, one cannot exclude the potential for a reconfiguration of the Vps75 tetramer upon histone binding which disfavours Cu (II) phenanthroline cross-linking at Vps75 K78C. In either case, these observations support the hypothesis that Vps75 tetramerisation restricts access to histone binding surfaces.

**Tetrameric Vps75 is incompatible with Rtt109 binding**

In addition to the histone chaperone function Vps75 also stimulates the catalysis of histone H3 acetylation when in complex with the histone acetyl transferase Rtt109 . Structural alignment between our tetrameric model of Vps75 in solution and crystal structures of the Vps75:Rtt109 complex , predicts the tetrameric form of Vps75 to be incompatible with Rtt109 binding (Figure 6A). We tested this incompatibility by PELDOR utilising the singly labelled Vps75 Y35RX2 dimer. If Rtt109 were to disrupt the Vps75 tetramer we would expect the modulation depth of the dipolar coupling to decrease as a function of Rtt109 concentration. Indeed, titrating in Rtt109 reduced the echo modulation depth of the doubly labelled Vps75 tetramer until, at a 2:1 (Vps75:Rtt109) stoichiometry, the resulting spectra represented only the exponential background decay of a single spin system (Figure 6B). This finding demonstrates that, in agreement with predictions from the model built of the Vps75 tetramer, Rtt109 binding to Vps75 is indeed incompatible with Vps75 in a tetrameric conformation. Thus Rtt109 binding obscures the Vps75 tetramerisation interface.

**The structurally related protein, Nap1, also forms tetramers**

SEC-MALS analysis of Nap1 at 150 mM sodium chloride was consistent with the dominant species in solution being tetrameric in equilibrium with a small proportion of Nap1 dimer with a weight averaged molecular mass of 187.7 kDa (theoretical tetramer 191.5 kDa). As predicted upon raising the salt concentration to 500 mM sodium chloride the Nap1 reverts to its dimeric form with a mass of 97.8 kDa (theoretical dimer 95.7 kDa) obtained from SEC-MALS analysis (Figure 7A). The previously reported Nap1 PPP mutant , in which three residues within the β-hairpin domain which protrude from the Nap1 earmuff domain are mutated to proline to inhibit secondary structure formation in this region (R301P, T302P, K305P), gives a mass of 98.4 kDa at 150 mM sodium chloride, indicating a predominantly dimeric (Figure 7B). This indicates that indeed the β -hairpins can influence tetramerisation. The concentration dependence of Nap1 self-association was assessed by bio-layer interferometry. Nap1 L120C dimers were specifically labelled with Maleimide-PEG2-Biotin and attached to Streptavidin biosensors. After washing at high salt and equilibrating at low salt, the association of unlabelled Nap1 was measured by bio-layer interferometry, and used to calculate a *K_d_* for tetramerisation of 3.57 μM (Figure 7CD).

**Probing the Nap1 tetramer structure using a four-spin system**

We initially attempted to devise a RX2 labelling strategy for labelling Nap1 at L120, the equivalent position to the Vps75 RX2 labelling site, however labelling at this site was inefficient (not shown). Upon further analysis, the Nap1 L120 site appeared to be more buried than its Vps75 counterpart, which may explain the poor cross-link labelling efficiency observed for this site. Due to the lack of a suitable RX2 labelling site a four spin system was used to further characterise the Nap1 tetramer. As the β-hairpins of opposing Nap1 dimers are implicated in the tetramerisation of Nap1 (Figure 7B), the sites T307 on the β-hairpin and E209 in close proximity to this region were chosen as labelling sites (Figure 8a). In addition the more distal site T251 was chosen to acquire additional restraints. PELDOR analysis of the three MTSL labelled cysteine mutants was performed under tetrameric (150 mM NaCl) and dimeric (500 mM NaCl) conditions. The background corrected dipolar evolution functions of all three labelling sites displayed the characteristic drop in oscillation depth associated with the increase in interacting spins in the system from two at 500 mM sodium chloride to four upon tetramerisation at 150 mM sodium chloride (Figure 8B).

As expected, upon tetramerisation short AB distances across the tetramerisation interface were observed in both Nap1 E209R1 and T307R1 distance distributions (Figure 8C). This is also apparent from the initial gradient of the dipolar oscillation becoming steeper at 150 mM compared to 500 mM sodium chloride (Figure 8B). However in the case of Nap1 T251R1 the initial gradient in the dipolar oscillation (Figure 8B) is indistinguishable at both 150 mM and 500 mM sodium chloride. This implies that the shortest distance in the Nap1 T251R1 tetramer is identical to the AB distance in the Nap1 T251R1 dimer and is consistent with the observation of two longer distances in the Nap1 T251R1 150 mM distance distribution, which were assigned as AC and AD (Figure 8C).

The globular domains of Nap1 are highly acidic and do not possess the charge complementarity observed in the Vps75 globular domains. As the β-hairpin extensions are highly basic and are required for tetramerisation, we investigated their conformation by introducing a labelling site at position T302. Consistent with the crystal structures of Nap1 dimers , these sites are separated by 10.2 nm at 500mM NaCl (Figure 8DE). To our knowledge this represents the longest distance measured using this approach.

At 150 mM NaCl a drop in oscillation depth was observed consistent with an increase in the number of interacting spins. Regularisation indicated the presence of at least three distance distributions (Figure 8E). There are several conformations the β-hairpin extensions could adopt to provide such a mixture of short and long distances (Figure 8F). While the measurements obtained support a rearrangement of the β-hairpins upon tetramerisation, they are not sufficiently robust to define the details of this reconfiguration.

In concert these observations are consistent with Nap1 tetramers having an arrangement in which the headphone fold domains are brought into close proximity. As a result it can be proposed that Nap1 like Vps75 adopts a tetrameric configuration at low salt concentrations, making it possible that the formation of tetrameric ring-like structures is a common feature of NAP-1 fold proteins.

**DISCUSSION**

The structure and function of Vps75 has been studied using a number of biochemical and biophysical techniques . Such studies have revealed the homo-dimeric nature of Vps75, but did not report the presence of tetramers. It is likely that the sensitivity of tetramerisation to ionic conditions contributes to this difference. The structural paralog of Vps75 in yeast is Nap1. Interestingly, Nap1 dimers have also been shown to self associate into higher molecular weight complexes , as have human Nap1 variants . Nap1 has been reported to form a number of higher order states ranging from tetramers to hexadecamers . These self association reactions are also sensitive to ionic conditions, and our own observations suggest that under conditions that are similar to those occurring within cells, the predominant form of Nap1 is that of a tetramer. It is unclear to us why these studies do not identify a dominant tetrameric species at moderate ionic conditions. One possibility is that over the longer time courses required for analytical ultracentrifugation oxidation of cysteine residues may have stabilised larger assemblies.

In a cellular context, a proportion of Vps75 is found stably associated with the histone acetyl transferase Rtt109. Interestingly, Rtt109 has been observed to form ring shaped complexes with Vps75 . However, there appears to be some plasticity in the interface between Vps75 and Rtt109 as both 2:1 and 2:2 Vps75:Rtt109 complexes have been reported. Furthermore, at first sight both complexes share a related ring-like organisation. In both cases the head-phone fold domains involved in Vps75 tetramerisation provide the Rtt109 interaction surfaces.. Nonetheless, unlike tetramerisation, the association of Rtt109 with Vps75 does not display sensitivity to high ionic conditions (data not shown) . This is most likely due to an additional, predominantly hydrophobic, contact between a loop region of Rtt109 and the globular fold of Vps75 . These additional contacts likely confer a higher affinity for Rtt109 in comparison to homotetramerisation consistent with our finding that Rtt109 can disrupt Vps75 tetramers (Figure 6). In addition, structure guided mutatgenesis indicates specificity in residues involved in the formation of homo and hetero oligomeric structures. For example, the double KK169EE mutation does not affect Rtt109 binding to Vps75 , but the single mutation K169E strongly reduced tetramer formation (Figure 5A). This provides scope for the quaternary interactions of Vps75 to be regulated independently and raises the question as to what function homotetermerisation might fulfil.

Tetramerisation of Vps75, restricts access to the central cavity that includes highly charged residues likely to be involved in histone binding. Just as positively charged histones require chaperones prior to incorporation into chromatin, the acidic surfaces of the chaperones themselves may require shielding when not occupied by their cargo as they may be prone to non specific association with basic proteins. Consistent with this concept, the majority of Vps75 purified from yeast lacks an interaction partner . In addition, while Vps75 protein levels remain constant through the cell cycle, both histones and Rtt109 are tightly regulated with a peak just prior to S-phase . As a result following the completion of DNA replication, the majority of Vps75 is not bound by either Rtt109 or histones. In this situation it is attractive to speculate that tetramerisation may act to restrict access to the acidic cavity formed at the interface between Vps75 dimers.

Our attempts to characterise the interaction of histones with Vps75 have been hindered by protein aggregation. As a result it is not clear whether histone binding dissociates Vps75 tetramers, or causes them to adopt an altered configuration. However, it is notable that when Nap1 is bound by histones, megadalton sized complexes have been observed. Cryo-Electron microscopy of these assemblies reveals they are made up of smaller particles with similar dimensions to the Vps75 tetramers we have characterised . We note that while a recent study indicates that Nap1 can bind histones H2A and H2B in a novel tetrameric conformation, this study does not distinguish whether Nap1 is present in the form of dimers or other oligomeric assemblies .

Structural information regarding the conformation of histone chaperones is crucial in deciphering the molecular mechanisms of nucleosome assembly and disassembly. In this study we demonstrate, using a number of in-solution methods, that the histone chaperones Vps75 and Nap1 adopt a homo-tetrameric structure. We propose that this may provide a self chaperoning function that serves to reduce non-specific interactions with the acidic surfaces that are encapsulated by the ring. Regulation of the oligomerisation of NAP-1 fold chaperones also potentially provides a means of regulating interactions with cargo proteins.

**Acknowledgments**

We would like to thank Govind Malwad for assistance in the mutagenesis of Vps75 and members of the DGN and TOH labs for valuable discussion. This work was supported by MRC grant G1100021 and Wellcome Senior Fellowship 095062.

**References**

1.	Laskey, R.A., Honda, B.M., Mills, A.D. and Finch, J.T. (1978) Nucleosomes are assembled by an acidic protein which binds histones and transfers them to DNA. *Nature (London)*, **275**, 416-420.

2.	Hansen, J.C., Nyborg, J.K., Luger, K. and Stargell, L.A. (2010) Histone Chaperones, Histone Acetylation, and the Fluidity of the Chromogenome. *J Cell Physiol*, **224**, 289-299.

3.	Ransom, M., Dennehey, B.K. and Tyler, J.K. (2010) Chaperoning Histones during DNA Replication and Repair. *Cell*, **140**, 183-195.

4.	Das, C., Tyler, J.K. and Churchill, M.E.A. (2010) The histone shuffle: histone chaperones in an energetic dance. *Trends Biochem Sci*, **35**, 476-489.

5.	Bonangelino, C.J., Chavez, E.M. and Bonifacino, J.S. (2002) Genomic screen for vacuolar protein sorting genes in Saccharomyces cerevisiae. *Molecular Biology of the Cell*, **13**, 2486-2501.

6.	Selth, L. and Svejstrup, J.Q. (2007) Vps75, a new yeast member of the NAP histone chaperone. *Journal of Biological Chemistry*, **282**, 12358-12362.

7.	Tsubota, T., Berndsen, C.E., Erkmann, J.A., Smith, C.L., Yang, L.H., Freitas, M.A., Denu, J.M. and Kaufman, P.D. (2007) Histone H3-K56 acetylation is catalyzed by histone chaperone-dependent complexes. *Molecular Cell*, **25**, 703-712.

8.	Park, Y.J., Sudhoff, K.B., Andrews, A.J., Stargell, L.A. and Luger, K. (2008) Histone chaperone specificity in Rtt109 activation. *Nature Structural & Molecular Biology*, **15**, 957-964.

9.	Krogan, N.J., Cagney, G., Yu, H.Y., Zhong, G.Q., Guo, X.H., Ignatchenko, A., Li, J., Pu, S.Y., Datta, N., Tikuisis, A.P. *et al.* (2006) Global landscape of protein complexes in the yeast Saccharomyces cerevisiae. *Nature*, **440**, 637-643.

10.	Kolonko, E.M., Albaugh, B.N., Lindner, S.E., Chen, Y., Satyshur, K.A., Arnold, K.M., Kaufman, P.D., Keck, J.L. and Denu, J.M. (2010) Catalytic activation of histone acetyltransferase Rtt109 by a histone chaperone. *Proceedings of the National Academy of Sciences of the United States of America*, **107**, 20275-20280.

11.	Fillingham, J., Recht, J., Silva, A.C., Suter, B., Emili, A., Stagljar, I., Krogan, N.J., Allis, C.D., Keogh, M.C. and Greenblatt, J.F. (2008) Chaperone control of the activity and specificity of the histone H3 acetyltransferase Rtt109. *Molecular and Cellular Biology*, **28**, 4342-4353.

12.	Selth, L.A., Lorch, Y., Ocampo-Hafalla, M.T., Mitter, R., Shales, M., Krogan, N.J., Kornberg, R.D. and Svejstrup, J.Q. (2009) An rtt109-independent role for vps75 in transcription-associated nucleosome dynamics. *Molecular and Cellular Biology*, **29**, 4220-4234.

13.	Xue, Y.M., Kowalska, A.K., Grabowska, K., Przybyt, K., Cichewicz, M.A., Del Rosario, B.C. and Pemberton, L.F. (2013) Histone Chaperones Nap1 and Vps75 Regulate Histone Acetylation during Transcription Elongation. *Molecular and Cellular Biology*, **33**, 1645-1656.

14.	Pramila, T., Wu, W., Miles, S., Noble, W.S. and Breeden, L.L. (2006) The Forkhead transcription factor Hcm1 regulates chromosome segregation genes and fills the S-phase gap in the transcriptional circuitry of the cell cycle. *Genes & Development*, **20**, 2266-2278.

15.	Berndsen, C.E., Tsubota, T., Lindner, S.E., Lee, S., Holton, J.M., Kaufman, P.D., Keck, J.L. and Denu, J.M. (2008) Molecular functions of the histone acetyltransferase chaperone complex Rtt109-Vps75. *Nature Structural and Molecular Biology*, **15**, 948-956.

16.	Tang, Y., Meeth, K., Jiang, E., Luo, C. and Marmorstein, R. (2008) Structure of Vps75 and implications for histone chaperone function. *Proceedings of the National Academy of Sciences of the United States of America*, **105**, 12206-12211.

17.	Park, Y.J. and Luger, K. (2006) The structure of nucleosome assembly protein 1. *Proceedings of the National Academy of Sciences of the United States of America*, **103**, 1248-1253.

18.	Su, D., Hu, Q., Zhou, H., Thompson, J.R., Xu, R.M., Zhang, Z. and Mer, G. (2011) Structure and histone binding properties of the Vps75-Rtt109 chaperone-lysine acetyltransferase complex. *J Biol Chem*, **286**, 15625-15629.

19.	Tang, Y., Holbert, M.A., Delgoshaie, N., Wurtele, H., Guillemette, B., Meeth, K., Yuan, H., Drogaris, P., Lee, E.H., Durette, C. *et al.* (2011) Structure of the Rtt109-AcCoA/Vps75 complex and implications for chaperone-mediated histone acetylation. *Structure*, **19**, 221-231.

20.	D'Arcy, S. and Luger, K. (2011) Understanding histone acetyltransferase Rtt109 structure and function: how many chaperones does it take? *Current Opinion in Structural Biology*, **21**, 728-734.

21.	Ishimi, Y. and Kikuchi, A. (1991) Identification and molecular cloning of yeast homolog of nucleosome assembly protein I which facilitates nucleosome assembly in vitro. *J. Biol. Chem.*, **266**, 7025-7029.

22.	Andrews, A.J., Downing, G., Brown, K., Park, Y.J. and Luger, K. (2008) A Thermodynamic Model for Nap1-Histone Interactions. *Journal of Biological Chemistry*, **283**, 32412-32418.

23.	Del Rosario, B.C. and Pemberton, L.F. (2008) Nap1 links transcription elongation, chromatin assembly, and messenger RNP complex biogenesis. *Molecular and Cellular Biology*, **28**, 2113-2124.

24.	Ohkuni, K., Shirahige, K. and Kikuchi, A. (2003) Genome-wide expression analysis of NAP1 in Saccharomyces cerevisiae. *Biochem Bioph Res Co*, **306**, 5-9.

25.	Kuryan, B.G., Kim, J., Tran, N.N.H., Lombardo, S.R., Venkatesh, S., Workman, J.L. and Carey, M. (2012) Histone density is maintained during transcription mediated by the chromatin remodeler RSC and histone chaperone NAP1 in vitro. *Proceedings of the National Academy of Sciences of the United States of America*, **109**, 1931-1936.

26.	McBryant, S.J. and Peersen, O.B. (2004) Self-association of the yeast nucleosome assembly protein 1. *Biochemistry-Us*, **43**, 10592-10599.

27.	Toth, K.F., Mazurkiewicz, J. and Rippe, K. (2005) Association states of nucleosome assembly protein 1 and its complexes with histones. *Journal of Biological Chemistry*, **280**, 15690-15699.

28.	Newman, E.R., Kneale, G.G., Ravelli, R.B.G., Karuppasamy, M., Nejadasl, F.K., Taylor, I.A. and McGeehan, J.E. (2012) Large Multimeric Assemblies of Nucleosome Assembly Protein and Histones Revealed by Small-angle X-ray Scattering and Electron Microscopy. *Journal of Biological Chemistry*, **287**, 26657-26665.

29.	Bowman, A., Ward, R., Wiechens, N., Singh, V., El-Mkami, H., Norman, D.G. and Owen-Hughes, T. (2011) The Histone Chaperones Nap1 and Vps75 Bind Histones H3 and H4 in a Tetrameric Conformation. *Molecular Cell*, **41**, 398-408.

30.	Jeschke, G. and Polyhach, Y. (2007) Distance measurements on spin-labelled biomacromolecules by pulsed electron paramagnetic resonance. *Physical Chemistry Chemical Physics*, **9**, 1895-1910.

31.	Schwieters, C.D., Kuszewski, J.J., Tjandra, N. and Clore, G.M. (2003) The Xplor-NIH NMR molecular structure determination package. *Journal of Magnetic Resonance*, **160**, 65-73.

32.	Hagelueken, G., Ward, R., Naismith, J.H. and Schiemann, O. (2012) MtsslWizard: In Silico Spin-Labeling and Generation of Distance Distributions in PyMOL. *Appl Magn Reson*, **42**, 377-391.

33.	Roessle, M.W., Klaering, R., Ristau, U., Robrahn, B., Jahn, D., Gehrmann, T., Konarev, P., Round, A., Fiedler, S., Hermes, C. *et al.* (2007) Upgrade of the small-angle X-ray scattering beamline X33 at the European Molecular Biology Laboratory, Hamburg. *J Appl Crystallogr*, **40**, S190-S194.

34.	Konarev, P.V., Volkov, V.V., Sokolova, A.V., Koch, M.H.J. and Svergun, D.I. (2003) PRIMUS: a Windows PC-based system for small-angle scattering data analysis. *J Appl Crystallogr*, **36**, 1277-1282.

35.	Svergun, D.I. (1992) Determination of the Regularization Parameter in Indirect-Transform Methods Using Perceptual Criteria. *J Appl Crystallogr*, **25**, 495-503.

36.	Svergun, D., Barberato, C. and Koch, M.H.J. (1995) CRYSOL - A program to evaluate x-ray solution scattering of biological macromolecules from atomic coordinates. *J Appl Crystallogr*, **28**, 768-773.

37.	Petoukhov, M.V. and Svergun, D.I. (2005) Global rigid body modeling of macromolecular complexes against small-angle scattering data. *Biophysical Journal*, **89**, 1237-1250.

38.	Fleissner, M.R., Bridges, M.D., Brooks, E.K., Cascio, D., Kalai, T., Hideg, K. and Hubbell, W.L. (2011) Structure and dynamics of a conformationally constrained nitroxide side chain and applications in EPR spectroscopy. *Proceedings of the National Academy of Sciences of the United States of America*, **108**, 16241-16246.

39.	Ward, R., Bowman, A., Sozudogru, E., El-Mkami, H., Owen-Hughes, T. and Norman, D.G. (2010) EPR distance measurements in deuterated proteins. *Journal of Magnetic Resonance*, **207**, 164-167.

40.	Milov, A.D., Ponomarev, A.B. and Tsvetkov, Y.D. (1984) Electron-electron double resonance in electron spin echo: Model biradical systems and the sensitized photolysis of decalin. *Phys. Lett*, **110**, 67-72.

41.	McBryant, S.J., Park, Y.J., Abernathy, S.M., Laybourn, P.J., Nyborg, J.K. and Luger, K. (2003) Preferential binding of the histone (H3-H4)2 tetramer by NAP1 is mediated by the amino-terminal histone tails. *J Biol Chem*, **278**, 44574-44583.

42.	Noda, M., Uchiyama, S., McKay, A.R., Morimoto, A., Misawa, S., Yoshida, A., Shimahara, H., Takinowaki, H., Nakamura, S., Kobayashi, Y. *et al.* (2011) Assembly states of the nucleosome assembly protein 1 (NAP-1) revealed by sedimentation velocity and non-denaturing MS. *Biochem J*, **436**, 101-112.

43.	Driscoll, R., Hudson, A. and Jackson, S.P. (2007) Yeast Rtt109 promotes genome stability by acetylating histone H3 on lysine 56. *Science*, **315**, 649-652.

44.	D'Arcy, S., Martin, K.W., Panchenko, T., Chen, X., Bergeron, S., Stargell, L.A., Black, B.E. and Luger, K. (2013) Chaperone Nap1 shields histone surfaces used in a nucleosome and can put H2A-H2B in an unconventional tetrameric form. *Mol Cell*, **51**, 662-677.

**Figure Legends**

**Figure 1. Size exclusion chromatography and multi angle light scattering analysis of Vps75 suggests it forms a stable tetramer under physiological conditions.** (A) SEC-MALS elution profiles of full length Vps75 in 150 mM (blue) and 500 mM (red) sodium chloride. The molar mass over elution peaks are shown in corresponding colours. (B) As in (A) except Vps75 lacking the unstructured C-terminal domain was analysed (residues 1-223). The association of soluble Vps75 with Vps75 dimers immobilised to a probe via a biotin linkage was measured using bio-layer interferometry. Changes in the response units were measured in the presence of increasing concentrations of soluble Vps75 dimers (C). A fit to the binding response data was then used to calculate the dissociation constant, *K_d_* (D).

**Figure 2. Probing the structure of the Vps75 tetramer through site-directed crosslinking spin labelling.** (A) The position of Y35RX2 in the Vps75 dimer. The boxed expansion shows a possible conformation of the crosslinked spin-labelled residue RX2 at position Y35 determined from molecular dynamic simulations. Monomers are differentially coloured blue and green for clarity. (B) Analysis of site-directed crosslink spin labelling. The compound 3,4-Bis-MTSL was titrated against a constant concentration of Vps75. Crosslinking efficiency was determined by resolving crosslinked from uncrosslinked species through separation by SDS-PAGE and quantification of coomassie staining (gel shown in inset). An arrow head indicates a 1:1 ratio of crosslinker to Vps75 dimer. (C) Schematic representation of three possible models for the tetramerisation of Vps75. Alternative dimers are shown in green and blue. Stars represent the approximate location of the spin-labelled side chain Y35RX2. PELDOR analysis of Vps75 Y35RX2 at 150 mM NaCl, raw dipolar evolution plot with background fit (D), background corrected dipolar evolution (E) and corresponding distance distribution (F) for Y35RX2. The modal distance between Y35RX2 residues is 7.8 nm.

**Figure 3. Probing the structure of the Vps75 tetramer using quadruply labelled complex.** (A) Schematic representation of the experimental approach for probing the Vps75 tetramer structure using a quadruply spin labelled system. In the highly ionic environment of 500 mM sodium chloride the dimeric form of Vps75 is prevalent, giving a single distance AB (left). At 150 mM sodium chloride the tetrameric form is prevalent, resulting in additional distances AC and AD (right). (B) Background corrected dipolar evolution for Vps75 E56R1 (left) and Vps75 K117R1 (right) under dimeric (red) and tetrameric (blue) conditions. (C) Distance distributions for Vps75 E56R1 (left) and Vps75 K117R1 (right) under dimeric (red) and tetrameric (blue) conditions. (D) A molecular model of the Vps75 tetramer was generated using the RX2 distance, and the . The two Vps75 dimers are differentially coloured green and blue for clarity. Left: the ‘Ring’ model with Y35RX2 and K117R1 labelling sites circled (E56R1 is omitted for clarity). The RX2-RX2’ distance is depicted as a black line, the AB distance is shown as a red line and AC and AD distances are shown as blue lines. Right: side view of the Vps75 tetramer with all labelling sites circled.

**Figure 4. SAXS analysis of Vps75 under tetrameric and dimeric conditions.** (A) Normalised distance distribution functions for Vps75 at 150 mM (blue, tetramer) and 500 mM NaCl (red, dimer). (B) Comparison of experimental SAXS curve (grey) with a CRYSOL calculated scattering curve (red) derived from a crystal structure of the dimer (PDB: 2ZD7), under high ionic conditions (500 mM sodium chloride). The crystal structure with unresolved residues modelled as a random coil is shown in the inset. (C) Comparison of the experimental SAXS curve (grey) under 150 mM sodium chloride, with a CRYSOL calculated curve of the Vps75 tetramer model (blue). The inset shows the modelled Vps75 tetramer with unresolved residues added in a random coil conformation. One dimer is shown in green, the other in blue. χ^2^ values for each fit are shown.

**Figure 5. Structure based mutagenesis to disrupt the Vps75 tetramer. (**A) Model of Vps75 tetramer, opposing Vps75 dimers in blue and green, with the positions of non-tetramerising mutants shown as colour coded spheres matching SEC-MALS elution profiles of the corresponding mutant; K169E (grey), K170E (red) and R164D (black) compared to wild type Vps75 (blue). (B) The location of K78 (yellow spheres) which was mutated to cysteine for disulphide cross-link formation to trap Vps75 in its tetrameric conformation and SEC-MALS analysis at 500 mM NaCl of Vps75 disulphide cross-linked at 150 mM NaCl via K78C.

**Figure 6. Vps75 tetramerisation is disrupted by binding of Rtt109.** (A) The tetrameric model of Vps75 in solution aligned to the crystal structure of the Vps75:Rtt109 complex (PDB: 3Q68). Vps75 dimers from the tetrameric model are represented in cartoon format and differentially coloured green and blue. The Vps75:Rtt109 crystal structure was aligned to the green Vps75 dimer of the tetrameric model. The surface of Rtt109 from the crystal structure is shown in red, whereas the surface of Vps75 from the crystal structure is omitted for clarity. (B) Background corrected dipolar evolutions from 100 μM Vps75 Y35RX2 dimer in the presence of Rtt109 at a ratio of 2:0, 2:0.25, 2:0.5 and 2:1, Vps75:Rtt109 as indicated. The reduction in the drop in spin echo in the presence of increasing Rtt109 indicates a transition from all spin-label-pairs to no spin-label-pairs in the sample, which is consistent with the conversion of double labelled tetrameric Vps75 to single labelled dimeric Vps75 associated with Rtt109.

**Figure 7. SEC-MALS analysis of the structurally related protein Nap1 suggests it also adopts a stable tetramer mediated by the β-hairpin.** (A) SEC-MALS elution profiles of Nap1 at 150 mM (blue) and 500 mM NaCl (red). (B) SEC-MALS analysis of the Nap1 PPP mutant. The molar mass over elution peaks are shown in corresponding colours. The association of soluble Nap1 with Nap1 dimers immobilised to a probe via a biotin linkage was measured using bio-layer interferometry. Changes in the response units were measured in the presence of increasing concentrations of soluble Nap1 dimers (C). A fit to the binding response data was then used to calculate the dissociation constant, *K_d_* (D).

**Figure 8. Probing the structure of the Nap1 tetramer using a quadruple spin system.** (A) Schematic representation of the experimental approach for probing the Nap1 tetramer structure using a quadruply spin labelled system. In 500 mM sodium chloride (red) the dimeric form of Nap1 is prevalent, giving a single distance AB (left). In 150 mM sodium chloride the tetrameric form is prevalent, resulting in additional distances AC and AD (right). (B) Corrected dipolar evolution for Nap1 E209R1 (left), Nap1 T251R1 (middle) and Nap1 T307R1 (right) under 500 mM NaCl (red) and 150mM NaCl (blue) conditions. (C) Distance distributions for Nap1 E209R1 (left), Nap1 T251R1 (middle) and Nap1 T307R1 (right) in 500mM NaCl (red) and 150 mM NaCl (blue). Corrected dipolar evolution (D) and distance distributions (E) for Nap1 T302R1. If the β-hairpin extensions did not rearrange upon tetramerisation as indicated schematically in (F) top panels a long and short distance would be anticipated. Reconfiguration of the β-hairpin’s as indicated in the bottom panels would provide a closer fit to the observed measurement’s. However, distances obtained from such a complex multi spin system are not sufficiently robust to distinguish between these.
